# Negative Posttrauma Cognitions Mediate the Association Between Morally Injurious Events and Trauma‐Related Psychopathology in Treatment‐Seeking Veterans

**DOI:** 10.1002/jts.22234

**Published:** 2017-11-15

**Authors:** Philip Held, Brian J. Klassen, Denise S. Zou, Blake S. Schroedter, Niranjan S. Karnik, Mark H. Pollack, Alyson K. Zalta

**Affiliations:** ^1^ Department of Psychiatry Rush University Medical Center Chicago Illinois USA; ^2^ Department of Behavioral Sciences Rush University Medical Center Chicago Illinois USA

## Abstract

Exposure to potentially morally injurious events has been shown to be associated with posttraumatic stress disorder (PTSD) and depression symptoms in military personnel. Few studies have examined factors that help to explain how potentially morally injurious events may contribute to the development of trauma‐related psychopathology. Negative posttrauma cognitions are thought to play a role in the etiology of PTSD and depression following trauma; however, it is unclear whether more global beliefs about the self, others, and world play a role in the development of PTSD and depression due to morally injurious events. Using structural equation modeling, we tested whether morally injurious experiences were indirectly related to trauma‐related psychopathology (PTSD and depression) through negative posttrauma cognitions in a sample of veterans seeking treatment for PTSD. An indirect effects only model best fit the data and showed that morally injurious experiences, specifically perceived transgressions by oneself and perceived betrayal, were indirectly associated with trauma‐related psychopathology through negative posttrauma cognitions, β = .17; 95% CI [.04, .31] and β = .25; 95% CI [.11, .41], respectively. Our findings suggest that negative posttrauma cognitions may be an important mechanism linking exposure to morally injurious events and trauma‐related psychopathology.

## Resumen

LAS COGNICIONES POSTRAUMÁTICAS NEGATIVAS MEDIAN LA ASOCIACIÓN ENTRE EVENTOS MORALES PERJUDICIALES Y LA PSICOPATOLOGÍA RELACIONADA‐CON‐EL‐TRAUMA EN VETERANOS QUE BUSCAN TRATAMIENTO

Herida Moral, Cogniciones Negativas y Patología

Se ha demostrado que la exposición a eventos potencialmente perjudiciales desde el punto de vista moral está asociada con el Trastorno de Estrés Postraumático (TEPT) y los síntomas de depresión en el personal militar. Pocos estudios han examinado los factores que ayudan a explicar cómo los eventos potencialmente perjudiciales desde el punto de vista moral pueden

contribuir al desarrollo de la psicopatología relacionada con el trauma. Se cree que las cogniciones postraumáticas negativas desempeñan un papel en la etiología del TEPT y la depresión posterior al trauma. Sin embargo, aún no es claro si las creencias más globales sobre sí‐mismo, sobre otros, y sobre el mundo, juegan un papel en el desarrollo de TEPT y depresión debido a eventos moralmente perjudiciales. Usando modelos de ecuaciones estructurales probamos si las experiencias moralmente perjudiciales se relacionaban indirectamente con la psicopatología relacionada‐con‐el‐trauma (TEPT y depresión) a través de cogniciones postraumáticas negativas, en una muestra de veteranos que buscaban tratamiento para el TEPT. Los efectos indirectos se ajustaron mejor a los datos, y mostraron que las experiencias moralmente perjudiciales se asociaron indirectamente con la psicopatología relacionada‐con‐el‐trauma, a través de cogniciones postraumáticas negativas, específicamente las transgresiones percibidas por uno mismo y la traición percibida, β = 0,17; IC del 95% [0,04, 0,31] y β = 0,25; 95% IC [0,11, 0,41], respectivamente. Nuestros hallazgos sugieren que las cogniciones postraumáticas negativas pueden ser un importante mecanismo que vincula la exposición a eventos moralmente perjudiciales y la psicopatología relacionada‐con‐el‐trauma.

## 抽象

Negative Posttrauma Cognitions Mediate the Relationship Between Morally Injurious Events and Trauma‐Related Psychopathology in Treatment‐Seeking Veterans

Traditional Chinese

標題: 尋求PTSD治療的退役軍人中༌負面的創傷後認知在不道德事件和創傷相關心理疾病的關係裡有中介效應

撮要: 過往研究顯示༌軍人經歷潛在不道德事件༌跟他們的創傷後壓力症(PTSD)和抑鬱症狀有關。可是༌少有研究檢視潛在不道德事件如何影響創傷相關的心理疾病發展。我們一般認為負面的創傷後認知對創傷後的PTSD與抑鬱症病源學有所影響༌但我們不清楚個人對自己、他人和世界的宏觀信念༌在個人經歷不道德事件時༌是否對PTSD與抑鬱症發展有所影響。本研究樣本為尋求PTSD治療的退役軍人。我們利用結構方程模型༌測試不道德事件經歷會否透過負面的創傷後認知༌而跟創傷相關的心理疾病༈PTSD與抑鬱症༉有間接關連。我們發現一個只有間接效應的模型與數據最吻合。它顯示不道德事件經歷༌特別是個人犯法的感知和背叛感知༌透過負面的創傷後認知༌跟創傷相關的心理疾病有間接關連༈分別為β = .17; 95% CI [.04, .31] 與β = .25; 95% CI [.11, .41]༉。結果反映༌負面的創傷後認知可能是個重要機制༌使不道德事件經歷跟創傷相關心理疾病有所關連

Simplified Chinese

标题: 寻求PTSD治疗的退役军人中༌负面的创伤后认知在不道德事件和创伤相关心理疾病的关系里有中介效应

撮要: 过往研究显示༌军人经历潜在不道德事件༌跟他们的创伤后压力症(PTSD)和抑郁症状有关。可是༌少有研究检视潜在不道德事件如何影响创伤相关的心理疾病发展。我们一般认为负面的创伤后认知对创伤后的PTSD与抑郁症病源学有所影响༌但我们不清楚个人对自己、他人和世界的宏观信念༌在个人经历不道德事件时༌是否对PTSD与抑郁症发展有所影响。本研究样本为寻求PTSD治疗的退役军人。我们利用结构方程模型༌测试不道德事件经历会否透过负面的创伤后认知༌而跟创伤相关的心理疾病༈PTSD与抑郁症༉有间接关连。我们发现一个只有间接效应的模型与数据最吻合。它显示不道德事件经历༌特别是个人犯法的感知和背叛感知༌透过负面的创伤后认知༌跟创伤相关的心理疾病有间接关连༈分别为β = .17; 95% CI [.04, .31] 与β = .25; 95% CI [.11, .41]༉。结果反映༌负面的创伤后认知可能是个重要机制༌使不道德事件经历跟创伤相关心理疾病有所关连

Morally injurious events involve “perpetrating, failing to prevent, bearing witness to, or learning about acts that transgress deeply held moral beliefs and expectations” (Litz et al., 2009, p. 700). Some common examples of potentially morally injurious situations include injuring or killing civilians, disproportionate violence, exposure to the aftermath of violence, violence within ranks, and betrayal (Litz et al., 2009; Stein et al., 2012). Evidence suggests that morally injurious events are experienced by a significant minority of service members; using a nationally representative sample of veterans, Wisco and colleagues (2017) reported that 10.8% of veterans endorsed personal transgressions, whereas 25.5% endorsed witnessing transgressions from others and experiencing betrayal from peers or leadership. Exposure to potentially morally injurious situations has been associated with posttraumatic stress disorder (PTSD) and depression symptoms (Bryan et al., 2016; MacNair, 2002; Nash et al., 2013). Despite the preliminary evidence suggesting that potentially morally injurious experiences predict PTSD and depression symptom severity, very few studies have examined the purported mechanisms by which moral injury can affect trauma‐related psychopathology (Frankfurt & Frazier, 2016).

There is evidence that negative posttrauma cognitions are a shared mechanism of PTSD and depression (Gonzalo, Kleim, Donaldson, Moorey, & Ehlers, 2012). In particular, negative, stable, internal, and global attributions (e.g., “The world isn't safe”) are central to the development of PTSD subsequent to a traumatic event (Foa, Ehlers, Clark, Tolin, & Orsillo, 1999), and the role such attributions play in the maintenance of depression is also well‐known (Abramson, Metalsky, & Alloy, 1989). Indeed, the presence of negative, stable, internal, and global attributions can differentiate between individuals with a diagnosis of PTSD (Foa et al., 1999) and depression (Ball, McGuffin, & Farmer, 2008), and those without. Investigators have described how moral injury involves negative stable, internal, and global attributions in response to the event (e.g., “I am a monster because of what I've done”; Drescher et al., 2011; Litz et al., 2009; Vargas, Hanson, Kraus, Drescher, & Foy, 2013). Moreover, Stein and colleagues (2012) found that exposure to the aftermath of violence (e.g., seeing dead bodies, grotesque images) was positively associated with greater levels of negative posttrauma cognitions about the world (e.g., “People can't be trusted”), providing further support for the association between morally injurious experiences and negative posttrauma cognitions. Given these findings, negative posttrauma cognitions may mediate the association between the experience of morally injurious events and trauma‐related psychopathology. Based on prior research, we hypothesized that morally injurious experiences would directly predict trauma‐related psychopathology and that negative posttrauma cognitions would mediate the association between potentially morally injurious experiences and trauma‐related psychopathology in a sample of treatment‐seeking veterans with PTSD.

## Method

### Participants and Procedure

All study procedures were approved by the Rush University Medical Center Institutional Review Board with a waiver of consent because all assessments were collected as part of routine clinical care procedures. The data for the present study were collected as part of the standard intake evaluation for veterans seeking mental health treatment at the Road Home Program, which provides mental health services to veterans and their family members free of charge, regardless of the extent of service, branch of service, or discharge status. Veterans in this study were enrolled in an intensive outpatient program (IOP); the primary eligibility criteria for this program included a primary diagnosis of PTSD based on the *Diagnostic and Statistical Manual of Mental Disorders* (5th ed.; *DSM‐5*; American Psychiatric Association, 2013) criteria, verified by the Clinician‐Administered PTSD Scale for *DSM‐5* (CAPS‐5; Weathers, Blake, et al., 2013). The present sample (*N* = 121) consisted of 66.1% males (*n* = 80) and 33.9% females (*n* = 41). The average age of participants in the sample was 39.38 years (*SD* = 9.27; range = 24 to 66). The majority of patients identified as White (66.9%) and Non‐Hispanic (75.2%), and served after the terrorist attacks of September 11, 2001 (88.4%), were deployed in support of recent operations in Iraq and Afghanistan (82.5%), were midlevel enlisted (E4‐E9; 76.9%), were honorably discharged (66.1%), and served in the Army (65.3%).

### Measures

#### Moral Injury Events Scale (MIES; Nash et al., 2013)

The MIES is a 9‐item measure of exposure to potentially morally injurious events. Studies that have examined the factor structure of the MIES have suggested a three‐factor solution (Bryan et al., 2016): Perceived Transgressions by Others (two items; e.g., “I am troubled by having witnessed others’ immoral acts”); Perceived Transgressions by Self (four items; e.g., “I acted in ways that violated my own moral code or values”); and Perceived Betrayals (three items; e.g., “I feel betrayed by leaders who I once trusted”). Items are scored from 1 (*strongly disagree*) to 6 (*strongly agree*); higher scores on the MIES subscales indicate a greater degree of exposure to potentially morally injurious events. Each MIES subscale has demonstrated satisfactory reliability and construct validity in active duty Marines (Nash et al., 2013) and other military samples (Bryan et al., 2016). The MIES subscales Perceived Transgressions by Others, Perceived Transgressions by Self, and Perceived Betrayals had very high internal consistency (Cronbach's αs = .81, .90, and .79, respectively).

#### Posttraumatic Cognitions Inventory (PTCI; Foa et al., 1999)

The PTCI is a 33‐item self‐report scale designed to measure maladaptive beliefs that have developed after exposure to a traumatic event, such as self‐blame, negative beliefs about self, and negative beliefs about others and the world. Items are scored from 1 (*totally disagree*) to 7 (*totally agree*); higher scores on the PTCI subscales indicate stronger negative posttrauma cognitions. The PTCI has demonstrated strong psychometric properties (Foa et al., 1999), and is regularly used to assess military populations with PTSD (e.g., Raab, Mackintosh, Gros, & Morland, 2015). The self‐blame, negative beliefs about self, and negative beliefs about others and the world subscales on the PTCI had high to very high internal consistency (Cronbach's αs = .91, .79, and .70, respectively).

#### PTSD Checklist for *DSM‐5* (PCL‐5; Weathers, Litz, et al., 2013)

Trauma‐related psychopathology was assessed using three measures, including the PCL‐5. The PCL‐5 is a 20‐item self‐report measure of PTSD symptom severity over the previous month. Respondents were asked to rate their symptoms from 0 (*not at all*) to 4 (*extremely*) in relation to their index trauma, with higher scores indicating greater PTSD severity. The PCL‐5 has been shown to be a reliable and valid measure of PTSD symptom severity in veteran and military populations (Bovin et al., 2015). The total PTSD scale on the PCL had very high internal consistency (Cronbach's α = .91).

#### Clinician‐Administered PTSD Scale for DSM‐5 (CAPS‐5; Weathers, Blake, et al., 2013)

The CAPS‐5 is a standardized interview designed to assess PTSD symptom severity in the past month according to the *DSM‐5* criteria. Clinicians rate each symptom based on specific behavioral anchors on a scale ranging from 0 (*absent*) to *3* (*extreme/incapacitating*) with higher scores reflecting more severe PTSD symptoms. Because a PTSD diagnosis was a requirement for admission into the IOP program, all veterans in this sample scored above the clinical cutoff. The total PTSD scale on the CAPS‐5 had very high internal consistency (Cronbach's α = .90).

#### Patient Health Questionnaire‐9 (PHQ‐9; Kroenke, Spitzer, & Williams, 2001)

The PHQ‐9 is a widely used 9‐item measure of self‐reported depression symptoms occurring in the previous 2 weeks. Items are rated from 0 (*not at all*) to 3 (*nearly every day*) and summed to create a total score, with higher scores reflecting greater severity of depression. In this sample, 85.9% of the veterans scored in the moderate to severe depression range. The total depression scale on the PHQ‐9 had high internal consistency (Cronbach's α = .80).

### Data Analysis

We first examined bivariate correlations of all study variables (displayed in Table 1). Next, we examined potential covariates (i.e., age, gender, education level, service era, military branch, and discharge status) that we suspected to potentially impact the outcome variables. None of the potential covariates were significantly correlated with the outcome variables and were therefore not included in the model. We then proceeded to develop a structural equation model using AMOS 24 with 10,000 bootstrap samples and 95% bias corrected confidence intervals to determine whether different morally injurious experiences were related to trauma‐related psychopathology through negative posttrauma cognitions. To generate bootstrap samples, we only included veterans who completed all PTSD and depression assessments; 17 veterans who did not complete the CAPS‐5 because it was not part of the standard clinical procedures at the time were excluded from our analyses. The software suite used to gather the self‐report instruments prevented veterans from submitting incomplete assessments. Thus, there was no missing self‐report assessment data, including demographic information; there was also no missing CAPS‐5 data. The model was estimated with maximum likelihood.

**Table 1:** Descriptive Statistics and Bivariate Correlations for Study Variables

Variable	*M*	*SD*	Range	Possible Range	Cronbach's α	1	2	3	4	5	6	7	8
1. MIES: Perceived Transgressions by Self	14.95	6.96	4–24	4–24	.90	—							
2. MIES: Perceived Transgressions by Others	9.77	2.98	2–12	2–12	.81	.49**	—						
3. MIES: Perceived Betrayal	12.63	5.11	3–18	3–18	.79	.22*	.40**	—					
4. PCL‐5	59.13	10.49	34–80	0–80	.86	.09	.06	.18*	—				
5. CAPS‐5	48.66	8.52	32–72	0–80	.81	.13	.16	.12	.34**	—			
6. PHQ‐9	17.83	4.96	5–27	0–27	.80	.04	.11	.16	.61**	.34**	—		
7. PTCI: Self‐Blame	21.89	6.53	5–35	5–35	.70	.26**	.20*	.37**	.56**	.30**	.40**	—	
8. PTCI: Negative Beliefs About Self	78.71	22.35	35–133	19–133	.91	.30**	.16	.35**	.54**	.27**	.46**	.86*	—
9. PTCI Negative Beliefs About Others/World	36.83	7.29	14–49	7–49	.79	.18*	.21*	.38**	.53**	.19*	.34**	.66**	.69**

*Note. N* = 121. MIES = Moral Injury Events Scale; PCL‐5 = PTSD Checklist for *DSM‐5*; CAPS‐5 = Clinician Administered PTSD Scale for *DSM‐5*; PHQ‐9 = Patient Health Questionnaire‐9; PTCI = Posttraumatic Cognitions Inventory; Cronbach's α = internal consistency reliability.

*p < .05. **p < .01.

**Figure 1:**
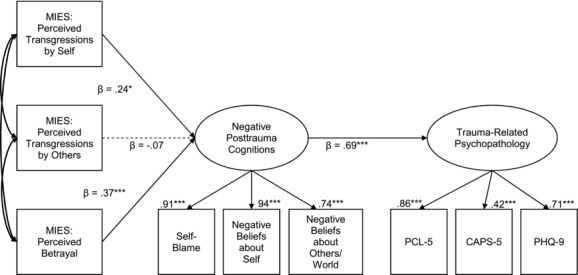
Indirect effects only model results. Model fit: χ^2^(23) = 26.07, *p* = .298; TLI = .99; SRMR = .04; CFI = .99; RMSEA = .03; AIC = 70.07; BIC = 131.57. Solid lines represent significant relationships at *p* < .05. Dashed lines represent nonsignificant relationships at *p* > .05. Numbers above the boxes are lambda values. MIES = Moral Injury Events Scale; PCL‐5 = PTSD Checklist for *DSM‐5*; CAPS‐5 = Clinician‐Administered PTSD Scale for *DSM‐5*; PHQ‐9 = Patient Health Questionnaire‐9. *p < .05. ***p < .001. TLI = Tucker‐Lewis index; SRMS = standardized root mean square residual; CFI = comparative fit index; RMSEA = root mean square error of approximation; AIC = Akaike information criterion; BIC = Bayesian information criterion.

The hypothesized measurement model included two latent variables: trauma‐related psychopathology and negative posttrauma cognitions. Trauma‐related psychopathology was indicated by the PCL‐5, CAPS‐5, and PHQ‐9. Negative posttrauma cognitions were indicated by the three PTCI subscales. PTCI subscales were not examined independently because of the high correlations between the subscales and lack of an a priori hypothesis that different morally injurious experiences would have unique associations with negative posttrauma cognitions. Therefore, we tested the more parsimonious model. The hypothesized structural model included direct paths from the MIES subscales to negative cognitions, a direct path from negative cognitions to trauma‐related psychopathology, and direct paths from the MIES subscales to trauma‐related psychopathology. Fit statistics were evaluated based on criteria suggested by Hu and Bentler (1999). Nonsignificant chi‐square values, root mean square error of approximation (RMSEA) values of .06 or lower, and comparative fit index (CFI) values greater than .95 indicate good fit (Hu & Bentler, 1999). Fit statistics suggested good overall fit of the measurement model, χ^2^(20) = 23.87, *p* = .248; Tucker‐Lewis Index (TLI) = .98; standardized root mean square residual (SRMR) = .03; CFI = .99; RMSEA = .04; Akaike information criterion (AIC) = 73.87; Bayesian information criterion (BIC) = 143.77. The model fit compared favorably to a *direct effects only* model in which the paths from the MIES to the negative posttrauma cognitions latent variable were set to zero, χ^2^(23) = 49.40, *p* = .001, TLI = .91, SRMR = .15, CFI = .94, RMSEA = .10, AIC = 93.40, BIC = 154.91. However, the *indirect effects only* model, in which the paths from the MIES to the trauma‐related psychopathology latent variable were set to zero, fit the data significantly better than the originally hypothesized model, χ^2^(23) = 26.07, *p* = .298, TLI = .99, SRMR = .04, CFI = .99, RMSEA = .03, AIC = 70.07, BIC = 131.57, BIC difference > 10 (cf. Raftery, 1995). Moreover, the correlations between the MIES subscales and trauma‐related psychopathology were small (*r*s = .06 to .18), indicating no meaningful associations between the MIES subscales and the outcome variables. Thus, we proceeded with the structural analysis using an *indirect effects only* model in which morally injurious experiences were indirectly related to trauma‐related psychopathology (PTSD and depression) through negative posttrauma cognitions (see Figure 1).

## Results

Standardized and unstandardized path estimates for the *indirect effects only* model are shown in Table 2. The model explained a medium amount of the variance in negative posttrauma cognitions (*R* ^2^ = .20) and a large amount of the variance in trauma‐related psychopathology (*R* ^2^ = .47). There were significant paths from perceived transgressions by self and perceived betrayal to negative posttrauma cognitions. There was also a significant path from negative cognitions to trauma‐related psychopathology. Based on Hayes's (2009) recommendation that indirect effects should be evaluated even when predictors and outcomes are not statistically significantly associated, we examined the indirect paths between the MIES subscales and trauma‐related psychopathology via negative posttrauma cognitions. The indirect effects of perceived transgressions by self, β = .17, 95% CI [0.04, 0.31], and perceived betrayal, β = .25, 95% CI [0.11, 0.41] to trauma‐related psychopathology via negative posttrauma cognitions were significant. There was no association between perceived transgressions by others and negative posttrauma cognitions.

**Table 2:** Unstandardized, Standardized, and Significance Levels for the Indirect Effects Only Model

Parameter Estimate	*B*	*SE*	β
MIES Perceived Transgressions by Self → Negative Posttrauma Cognitions	0.21	.08	.24*
MIES Perceived Transgressions by Others → Negative Posttrauma Cognitions	−0.14	.21	−.07
MIES Perceived Betrayal → Negative Posttrauma Cognitions	0.43	.11	.37***
Negative Posttrauma Cognitions→ Trauma‐Related Psychopathology	1.04	.14	.69***
Negative Posttrauma Cognitions→ Self‐Blame	1.00	N/A	.91
Negative Posttrauma Cognitions→ Negative Beliefs about Self	3.52	.23	.94***
Negative Posttrauma Cognitions→ Negative Beliefs About Others/World	0.90	.09	.74***
Trauma‐Related Psychopathology → PCL‐5	1.00	N/A	.86
Trauma‐Related Psychopathology → CAPS‐5	0.40	.09	.42***
Trauma‐Related Psychopathology → PHQ‐9	0.39	.06	.71***

*Note*. *N* = 121. MIES = Moral Injury Events Scale; PCL‐5 = PTSD Checklist for *DSM‐5*; CAPS‐5 = Clinician‐Administered PTSD Scale for *DSM‐5*; PHQ‐9 = Patient Health Questionnaire‐9.

*p < .05. ***p < .001.

## Discussion

The purpose of the present study was to examine the association between potentially morally injurious experiences, negative posttrauma cognitions, and trauma‐related psychopathology in a sample of treatment‐seeking veterans with PTSD. Perceived transgressions by oneself and perceived betrayal were only indirectly associated with trauma‐related psychopathology through negative posttrauma cognitions. These findings align with existing cognitive models of trauma, which attribute the genesis and maintenance of PTSD and depression symptoms, at least in part, to the development of negative posttrauma cognitions following traumatic experiences (Ehlers & Clark, 2000). In line with a recent case report (Held, Klassen, Brennan, & Zalta, 2017), the present findings suggest that individuals who perceive themselves to have engaged in moral transgressions are likely to blame themselves for what they have done and may view themselves more negatively. Similarly, individuals who perceive betrayal may develop more negative views of others and the world, as well as negatively evaluate themselves and their own actions. Our study is the first that demonstrated that these negative posttrauma cognitions appear to be an important mechanism by which morally injurious experiences lead to trauma‐related psychopathology.

To our surprise, perceived transgressions by others were not significantly associated with negative posttrauma cognitions in our model. One possible explanation could be that witnessing acts of moral transgression via commission or omission by others becomes problematic only if the individual who witnessed these transgressions assumes responsibility or blame (e.g., “I should have stopped this from happening”), begins to view themselves negatively because of what they have observed (e.g., “I am part of the same unit and therefore just as bad as they are”), or perceives these actions as betrayals by fellow service members or leaders whom they once trusted (e.g. “I should have been able to trust my brothers‐in‐arms”).

Inconsistent with some previous reports (e.g., Bryan et al., 2016; Nash et al., 2013), we did not observe a direct association between potentially morally injurious experiences and trauma‐related psychopathology, and an *indirect effects only* model fit the data best. Our population included a more severe clinical sample seeking intensive treatment; veterans in our sample also had higher average MIES scores (Perceived Transgressions by Others: 4.88, Perceived Transgressions by Oneself: 3.73, and Perceived Betrayals: 4.20) compared with Bryan and colleagues’ (2016) study (3.68, 2.49, and 3.21, respectively). It appears that among traumatized individuals with PTSD, the experience of morally injurious events may not significantly worsen PTSD and depression symptoms. Moreover, our findings suggest that among treatment‐seeking veterans, potentially morally injurious events do not predict PTSD and depression severity unless one engages in self‐blame and negative beliefs about themselves, others, or the world. Future studies should investigate and compare the association between morally injurious experiences and trauma‐related psychopathology in clinical and nonclinical samples.

Conclusions drawn from this study are limited by the cross‐sectional nature of the data, as well as by the small sample size. The cross‐sectional models of longitudinal processes both prevent inferences regarding causality and may provide biased estimates of effects between the constructs that may not reflect findings from longitudinal data (Maxwell & Cole, 2007). Therefore, it is essential that researchers collect longitudinal data regarding potentially morally injurious experiences and its sequelae. It is also important to note that the negative posttrauma cognitions reported by the veterans in this sample may not result directly from morally injurious experiences but may be the result of other traumas the veterans may have experienced. Further, given the overlap of negative posttrauma cognitions assessed by the PTCI and the new diagnostic criteria for PTSD according to the *DSM‐5*, the association between the PTCI and the PCL‐5 as well as CAPS‐5 may be mildly inflated. Lastly, it is important to consider that potentially morally injurious experiences are not synonymous with moral injury. Thus, conclusions drawn from this study may only apply to self‐reported experiences of potentially morally injurious events, but not the broader construct of moral injury.

In summary, the present study suggests that potentially morally injurious experiences and PTSD and depression severity appear to be only indirectly related through negative posttrauma cognitions in treatment‐seeking veterans with PTSD. Our findings may explain why a variety of treatment approaches, such as cognitive processing therapy (Resick, Monson, & Chard, 2014), prolonged exposure (Foa, Hembree, & Rothbaum, 2007), and adaptive disclosure (Gray et al., 2012; Litz, Lebowitz, Gray, & Nash, 2016), that target posttrauma cognitions have been found to be effective at reducing PTSD and depression symptoms in veterans affected by moral injury (Gray et al., 2012; Held et al., 2017; Litz et al., 2016). Future studies with veterans who have been exposed to potentially morally injurious events should examine both a more comprehensive model and the impact of reducing negative posttrauma cognitions over the course of treatment on PTSD and depression symptoms.
